# Revisiting the Mechanistic Pathway of Gas-Phase Reactions in InN MOVPE Through DFT Calculations

**DOI:** 10.3390/molecules30040971

**Published:** 2025-02-19

**Authors:** Xiaokun He, Nan Xu, Yuan Xue, Hong Zhang, Ran Zuo, Qian Xu

**Affiliations:** 1 Suzhou Institute of Technology, Jiangsu University of Science and Technology, Zhangjiagang 215600, China; kenhe25@163.com; 2 Institute for Energy Research, Jiangsu University, Zhenjiang 212013, China; 3 State Key Laboratory of High-Efficiency Utilization of Coal and Green Chemical Engineering, Faculty of Advanced Science and Technology, Ningxia University, Yinchuan 750021, China; nan.xu@nxu.edu.cn; 4 Department of Chemistry and Biochemistry, The University of Mississippi, Oxford, MS 38677, USA; 5 Department of Chemistry, The University of Akron, Akron, OH 44325, USA; 6 School of Energy and Power Engineering, Jiangsu University, Zhenjiang 212013, China; thornbird05@126.com (H.Z.); rzocean111@163.com (R.Z.)

**Keywords:** DFT, InN, gas-phase reactions, MOVPE, NBO, potential energy surface

## Abstract

III-nitrides are crucial materials for solar flow batteries due to their versatile properties. In contrast to the well-studied MOVPE reaction mechanism for AlN and GaN, few works report gas-phase mechanistic studies on the growth of InN. To better understand the reaction thermodynamics, this work revisited the gas-phase reactions involved in metal–organic vapor-phase epitaxy (abbreviated as MOVPE) growth of InN. Utilizing the M06-2X function in conjunction with Pople’s triple-ζ split-valence basis set with polarization functions, this work recharacterized all stationary points reported in previous literature and compared the differences between the structures and reaction energies. For the reaction pathways which do not include a transition state, rigorous constrained geometry optimizations were utilized to scan the PES connecting the reactants and products in adduct formation and XMIn (M, D, T) pyrolysis, confirming that there are no TSs in these pathways, which is in agreement with the previous findings. A comprehensive bonding analysis indicates that in TMIn:NH_3_, the In-N demonstrates strong coordinate bond characteristics, whereas in DMIn:NH_3_ and MMIn:NH_3_, the interactions between the Lewis acid and base fragments lean toward electrostatic attraction. Additionally, the NBO computations show that the H radical can facilitate the migration of electrons that are originally distributed between the In-C bonds in XMIn. Based on this finding, novel reaction pathways were also investigated. When the H radical approaches MMInNH_2_, MMIn:NH_3_ rather than MMInHNH_2_ will generate and this is followed by the elimination of CH_4_ via two parallel paths. Considering the abundance of H_2_ in the environment, this work also examines the reactions between H_2_ and XMIn. The Mulliken charge distributions indicated that intermolecular electron transfer mainly occurs between the In atom and N atom whiling forming (DMInNH_2_)_2_, whereas it predominately occurs between the In atom and the N atom intramolecularly when generating (DMInNH_2_)_3_.

## 1. Introduction

In recent decades, InN and its ternary alloy InGaN have garnered significant interest due to their low band gap energy (~0.6 ev), small effective mass of electrons, and high electron mobility. These properties position them as promising materials for various applications, including high-speed electronics, visible LED lights and solar flow batteries [1,2]. The primary technology employed for the preparation of InN and other III-nitride films is metal–organic vapor-phase epitaxy (abbreviated as MOVPE in further discussions) [3,4,5], which is favored for its scalability, high growth rate and excellent reproducibility.

MOVPE consists of two pivotal steps, and they are gas-phase reactions and surface reactions. Gas-phase reactions are crucial in determining the precursors for surface reactions, which, in turn, influence the growth rate and quality of films. Consequently, extensive research has been conducted to reveal the gas-phase reaction mechanisms of III-nitride films through the MOVPE process, particularly AlN and GaN [6,7,8,9,10]. And this paper concentrates on the gas-phase reaction mechanisms in InN growth specifically. It is recognized that there are two competing gas reaction paths during the growth of III-nitride films via MOVPE. The first pathway involves the pyrolysis of group-III precursors (typically TMX, X = Al, Ga and In) through a stepwise elimination of CH_3_ [6,8,10,11]. Utilizing a toluene carrier flow system, Jacko [12,13] studied the pyrolysis of TMX and concluded that the difficulty of pyrolysis follows the trend: TMX > MMX > DMX. Using a stagnation-point reactor, Hwang [14] investigated TMIn pyrolysis in an N_2_ atmosphere and found that TMIn pyrolysis occurs at a temperature range of 120–535 °C. However, these experiments could not accurately reflect the actual growth process due to the isolation of group-V precursor NH_3_. Mihopoulos [15] identified that mixing TMAl with NH_3_ can give rise to the instantaneous formation of a Lewis acid-base adduct, TMAl:NH_3_, which subsequently undergoes irreversible decomposition and releases CH_4_. This process is abbreviated as the “adduct path”, representing an alternative gas reaction pathway that is believed to initiate the formation of nanoparticles. Using in situ light scattering, Creighton [16] observed a layer of scattering by nanoparticles approximately 6 mm away from the substrate for both AlN, GaN and InN growth. In the case of AlN growth, the presence of CH_4_ was detected by FTIR measurements, whereas no CH_4_ was observed during the growth of GaN and InN. This discrepancy is attributed to the stronger Al-N bond compared to the Ga-N and In-N bonds. The stronger Al-N bond facilitates the formation of the amide DMAlNH_2_, accompanied by the elimination of CH_4_ through the irreversible decomposition of TMAl:NH_3_. In contrast, during the growth of GaN and InN, the adducts tend to undergo reversible dissociation back to TMGa or TMIn. Based on ab initio computations, Nakamura [17] concluded that the likelihood of CH_4_ elimination through the irreversible decomposition of adducts decreased down the periodic table and follows the trend: AlN > GaN ≈ InN. Agreeing with the theoretical investigations, experimental work [18] also suggests that the robust Al-N bond renders the irreversible decomposition of TMAl:NH_3_ as the predominant gas-phase reaction in AlN MOVPE growth. In addition to these two competing pathways, radicals may significantly influence how the gas-phase reaction progresses. In Creighton’s experimental study of GaN and InN growth [16], a switch in the carrier gas from H_2_ to N_2_ resulted in a notable decrease in scattering intensity from nanoparticles, while minima change was observed in AlN growth. This indicates that H radicals, which are produced from reactions involving H_2_, have a substantial impact on the growth of GaN and InN, whereas AlN is more inert to the radicals. Cavallotti [19] stated that both the pyrolysis path and the adduct path will be accelerated by the radicals, such as H produced by reactions between H_2_ and CH_3_ radicals.

The growth mechanisms of InN may differ significantly from those of AlN and GaN due to comparatively weaker bonds of In-N and In-C when contrasted with Al and Ga. However, despite the extensive studies on the growth mechanisms of AlN and GaN, very few gas-phase reaction mechanisms were targeted specifically on InN. Despite a systematic study on gas-phase reaction mechanisms in InN MOVPE growth from the thermodynamic and kinetic perspective performed using DFT calculations [18,20], the detailed bonding mechanisms of gas-phase reactions at the microscopic level are still unclear. To recalibrate the DFT baseline, all the gas-phase reactions of InN MOVPE proposed in previous research are revisited with a larger basis set in this study. The variations in energetics and kinetics were probed by comparing the benchmarking results reported herein with previous literature. Furthermore, all reactions reported herein will be investigated using the electrostatic potential (ESP), the natural bond orbital (NBO) and electron transfer to shed light on the detailed bonding mechanisms at the microscopic level.

## 2. Results and Discussions

### 2.1. Revisit the Reaction Pathways Proposed in the Literature

Built on previous work [20], all electronic structures optimized at the M06-2X/6-31G(d,p) level of theory were recharacterized with the 6-311G(d,p). As illustrated in Tables S1 and S2, most bond lengths differ by less than 0.5%, and all bond angles are within a 0.85% deviation based on the stationary points in all proposed reaction pathways documented in the literature [20]. As illustrated in Table 1, the thermodynamic aspects of *E*_a_ and ∆*H* are consistent with the results reported in previous work that incorporate a polarized double-ζ split-valence basis, as the most exothermic reactions are XMIn pyrolysis reactions with H radicals involved, i.e., R5–R5b, while the most endothermic reactions are XMIn pyrolysis reactions (P4–P4b). When comparing these results, the extrapolated reaction enthalpies are always within 1.2 kJ/mol deviation. Similarly, the irreversible decomposition reactions of the adducts (A2 and A2a) always demand the largest energy input to overcome the energy barrier, with the evaluated *E*_a_ differing no more than 1.3 kJ/mol. These slight variations indicate that in conjunction with the M06-2X function, computations with a double-ζ basis set can provide reliable predictions on reaction energetics relative to a larger basis set and hence could be an option to better balance between computation cost and accuracy.

Adduct formation marks the beginning of the adduct path, which plays a crucial role in the competitive processes involved in the growth of III-nitride semiconductor thin films. Following the formation of the adduct, it may either dissociate back into TMIn and NH_3_ via reaction A1 or follow an irreversible decomposition reaction to form the DMInNH_2_ and CH_4_ via reaction A2. On the other hand, TMIn can also react with NH_3_ to form DMInNH_2_ through a bimolecular interaction (A3). As shown in Table 1, the *E*_a_ of reactions A2, A2a, and A2b are 202.74 kJ/mol, 119.58 kJ/mol and 86.25 kJ/mol, respectively. The *E*_a_ of A2 is ca. 83 kJ/mol and 116 kJ/mol higher compared to A2a and A2b. In addition, A2 and A2a are endothermic reactions, while A2b is an exothermic reaction. The heat released by A2b can supply the necessary energy needed to overcome the barrier and facilitate the chemical process, thus promoting the occurrence of the reaction. In this case, it is reasonable to conclude that reaction A2 presents a greater challenge to occur compared to A2a and A2b.

Another competitive reaction path is the pyrolysis of XMIn (where X = M, D, T). According to the ∆*H* of P4–P4b presented in Table 1, the likelihood of occurrence follows the trend P4a > P4b > P4. To further understand the bonding strength between In and C, the dissociation energy (abbreviated as *BDE* in following discussion) of TMIn, DMIn, and MMIn was determined via Equation (2) in Section 3 and the magnitude of these three are 304.63 kJ/mol, 128.02 kJ/mol, and 238.08 kJ/mol, respectively, indicating the required energy to dissociate the In-N bond follows the trend TMIn > MMIn > DMIn. This trend aligns with the findings in previous experimental research reported by Jacko [13]. Interestingly, the In-C bond lengths are 2.15 Å for TMIn, 2.19 Å for DMIn, and 2.23 Å for MMIn, which appears to contradict the trend observed in *BDE* analyses. This discrepancy suggests that there might be underlying mechanisms influencing this phenomenon. To further elucidate the distinct covalent bonding characteristics in these three molecules, an NBO analysis was conducted, and the results will be illustrated in Section 2.2.1.

A previous DFT computational study indicated that, in addition to direct pyrolysis, XMIn can also react with H radicals and proceed with stepwise CH_4_ eliminations. These reactions are denoted as R5–R5b listed in Table 1. When the environment is free of H radicals, the pyrolysis reactions (P4–P4b) have positive Δ*G*s, suggesting that these are non-spontaneous processes. In contrast, with a negative Δ*G* for pyrolysis reactions involving H radicals, the processes are spontaneous. Obviously, H radicals play a role in promotion of XMIn pyrolysis. From the ∆*H* listed in Table 1, reactions P4–P4b are endothermic; however, with the aid of H radicals, pyrolysis reactions R5–R5b are exothermic provided they overcome the *E*_a_. The detailed bonding mechanism analysis of H radicals with XMIn is conducted in Section 2.2.2. Due to the high reactivity, H radicals can also react with amide and lead to CH_4_ elimination (R7–R9). In reaction R8, it is predicted that the H radical will interact with the In atom to yield MMInHNH_2_, given that both the In atom and the H radical have one unpaired electron. However, upon bringing the H radical near the N atom in MMInNH_2_, the unconstrained geometry optimization confirmed the minimum was the adduct, MMIn:NH_3_ rather than MMInHNH_2_. Denoted as R8a, the transformation from MMIn:NH_3_ to MMInHNH_2_ has a electronic energy difference ∆*E* of 6.13 kJ/mol, indicating that the product is more likely to be kept in the form of MMIn:NH_3_ while the interactions occur between the H radical and MMInNH_2_.

MMInNH_2_ + H→MMIn:NH_3_ (R8a)

MMIn:NH_3_→MMInHNH_2_ (R8b)

**Figure 1:**
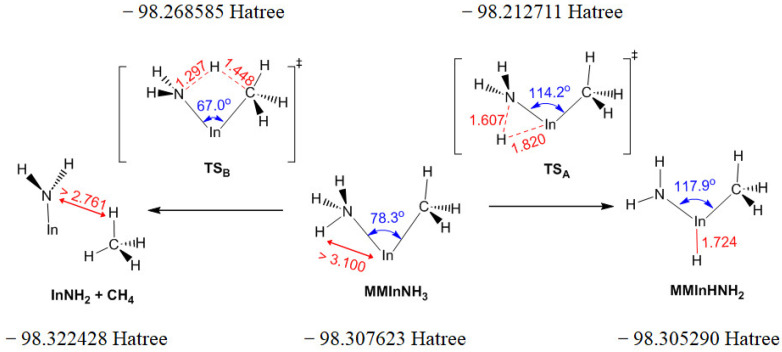
Two parallel paths with the elimination of CH_4_ from MMIn:NH_3_ and corresponding molecular structures.

Based on the two isomeric minima characterized above, a TS was characterized using the QST2 method and verified MMIn:NH_3_ as the reactant and MMInHNH_2_ as the product. According to the above discussion, there are two parallel paths to eliminate CH_4_ from MMIn:NH_3_, one is reaction A2b listed in Table 1 (also the left path in Figure 1) and the other is the conversion of MMIn:NH_3_ to MMInHNH_2_ with a *E*_a_ of 229.97 kJ/mol (denoted as (R8b)) followed by the reaction R9 with the elimination of CH_4_ and the above process is denoted as the “H-shift path” (the right path in Figure 1). These two parallel paths and corresponding molecular structures are also illustrated in Figure 1. Further investigations into the chemical bonding of this process will be revealed with ESP and NBO in Section 2.2.2. The left pathway involves only one TS (TS_B_ in Figure 1), whereas the path on the right needs to cross over two TSs (the TS_A_ in Figure 1 and the TS in reaction R9); therefore, the *E*_a_ of the left path (86.25 kJ/mol) is lower than that of the right path (229.97 kJ/mol and 93.62 kJ/mol) and the left path may have a higher priority. When searching for TS_A_, the energy of the product InNH_2_ and CH_4_ optimized as a pair is ca. 9 kJ/mol lower than the summed energy when they are optimized in isolation.

Because CH_3_ radicals that are produced from XMIn pyrolysis can also react with the H_2_ carrier gas in group III-nitride MOVPE and generate H radicals, the reaction pathways could be altered via either the pyrolysis path (R5–R5b) or the adduct path (R7, R8a, R8b and R9). However, as the H radicals can also merge into H_2_, along with the H_2_ brought in by the substantial flow rate of the carrier gas in the reaction chamber, a set of novel reaction pathways H12–H14 (H_2_-invovled paths in the following discussion), as listed in Table 2, will be further discussed in Section 2.2.3.

### 2.2. NBO and ESP Analysis

#### 2.2.1. Two Typical Competitive Routes in the InN MOVPE Process

**Figure 2:**
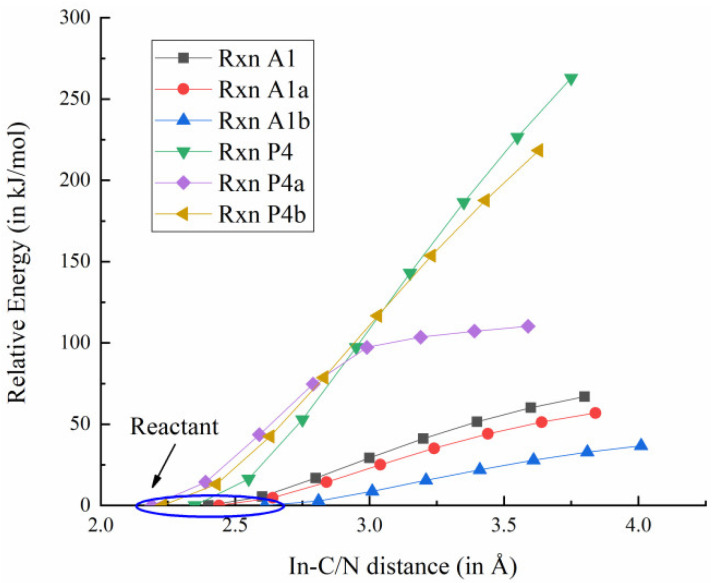
The relaxed scan for adduct formation (A1–A1b) and pyrolysis reaction (P4–P4b). [Annotation 1] The PES was explored by constrained geometry optimization, and connects the dissociated In(CH_3_)_x−1_ and CH_3_ or In(CH_3_)_x_ and NH_3_. [Annotation 2] Relative Energy refers to the electron energy difference between the scan points and the 1st scan point (i.e., reactants).

The adduct path represents a crucial competitive mechanism in the MOVPE process of III-nitrides, as the interaction between TMIn and NH_3_ can trigger a cascade of reactions that ultimately generate nanoparticles. Other than TMIn:NH_3_, similar reactions can occur with DMIn and MMIn [20], which are denoted as reactions A1, A1a and A1b in Table 1. It is anticipated that the rapid formation of the TMIn:NH_3_ adduct can process upon the collision of TMIn with NH_3_ without involving a TS [18]. To verify this interpretation and probe surface between the reactants and products in reactions A1, A1a and A1b, constrained geometry optimizations were employed to simulate the In-N bond formation and obtain reliable electronic energy. Take reaction A1 as an example—the In in TMIn and the N in NH_3_ were constrained as 3.8 Å apart, while the rest of the geometry parameters were relaxed through geometry optimization. This procedure was repeated to decrease the In-N distance from 3.6 Å to 2.4 Å, with a step size decrement of 0.2 Å. As illustrated in Figure 2, the monotonic change in electronic energy indicates that the rapid addition between a Lewis acid and base pair always occurs in one concerted step, which is consistent with previous research results [18].

**Figure 3:**
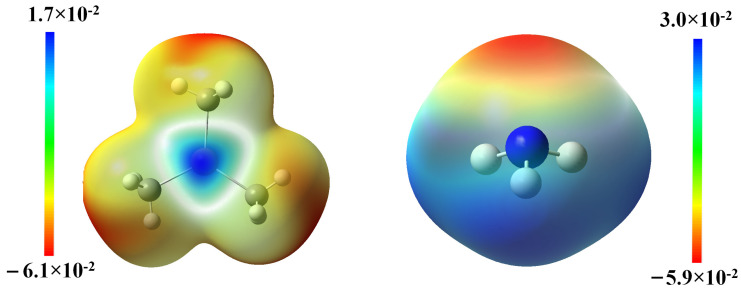
The ESP map of TMIn and NH_3_.

To gain insight into the electronic distributions among the pivotal structures in reaction A1, the ESP of the two reactants was mapped and illustrated in Figure 3. The results confirm that the electron-proficient N atom in NH_3_ might initiate a nucleophilic attack to the electron-deficient In center in TMIn and facilitate the formation of the dative In-N bond in TMIn:NH_3_.The summarized NBO analysis demonstrated in Table 3 indicates that the In-N bond consists of 5% electronic character from In and 95% from N. This finding further substantiates the notion that the non-bonding electron pair from N could migrate into the vacant valence orbitals of In, thereby facilitating the formation of a coordinate bond.

The absences of TSs in reactions A1, A1a and A1b suggest that these reactions might proceed with similar mechanisms. To further substantiate this hypothesis, NBO analyses were conducted to probe the more detailed electron transfers in reactions A1a and A1b and the summarized results are illustrated in Tables S3 and S4. NBO analysis indicated that no bond was formed between In and N atoms in both DMIn:NH_3_ and MMIn:NH_3_, which is quite different from that of TMIn:NH_3_. From the perspective of In-N bond length, the In-N bond in DMIn:NH_3_ (2.44 Å) and especially in MMIn:NH_3_ (2.61 Å) differs distinctly from TMIn:NH_3_ (2.40 Å). Thus, qualitatively, the bonding mechanism of these adducts may be essentially different. To further verify the interactions between MMIn/DMIn and NH_3_, we extrapolated the interaction energy (abbreviated as *E*_int_ in following discussion) between the XMIn (X = M, D, T) and NH_3_ in TMIn:NH_3_, DMIn:NH_3_, and MMIn:NH_3_ via Equation (3) in Section 3. The results suggest that TMIn exhibits the highest *E*_int_ with NH_3_, and it is ca. 20 and ca. 46 kJ/mol higher compared with DMIn and MMIn. Based on the *E*_int_, the *BDE* values of them are also computed to further verify the bond strength between In and N in TMIn:NH_3_, DMIn:NH_3_, and MMIn:NH_3_. The *BDE* values of TMIn:NH_3_, DMIn:NH_3_, and MMIn:NH_3_ are 92.76 kJ/mol, 80.02 kJ/mol, and 54.05 kJ/mol, respectively. Additionally, the *BDE* values of the In-N bond for DMInNH_2_ and MMIn(NH_2_)_2_ are determined to be 381.61 kJ/mol and 370.57 kJ/mol, respectively, at the same computational level. These results are much larger than that for TMIn:NH_3_, DMIn:NH_3_, and MMIn:NH_3_.

In addition, the Mayer bond order between In and N was verified via Multiwfn [21] in five pivotal structures, XMIn(NH_2_)_Y_ (where X = M, D, T and Y = 1 or 2). Listed with *BDE* values in Table 4, the Mayer bond orders of the In-N bond are 0.205, 0.198, 0.142, 0.91, and 0.85 in TMIn:NH_3_, DMIn:NH_3_, MMIn:NH_3_, DMInNH_2_, and MMIn(NH_2_)_2_, respectively. From the perspective of the NBO and Mayer bond order results, the In-N bond in DMInNH_2_, MMIn(NH_2_)_2_ is a single bond, while in TMIn:NH_3_, it is a single coordinate bond. Since NBO shows no bond between In and N for DMIn:NH_3_, MMIn:NH_3_ and the In-N bond orders of these two are lower than in TMIn:NH_3_; thus, we suspect that the interaction between In and N in DMIn:NH_3_ and MMIn:NH_3_ results from electrostatic attraction.

To further probe the charge distributions between In and N in XMIn:NH_3_ (where X = M, D and T), the Mulliken charge transfer was assessed. For a Lewis adduct AB which consists of the acid (A) and base (B), the charge transferred from segment B to segment A (*N*_B→A_) is determined using Equation (1), where *N*_B,P_ and *N*_B,R_ are the Mulliken charge of segment B in the product and reactant, respectively.

*N*_B→A_ = *N*_B,P_ − *N*_B,R_ (1)

As a result, the *N*_B→A_ in TMIn:NH_3_, DMIn:NH_3_, and MMIn:NH_3_ is 0.126 a.u., 0.122 a.u., and 0.082 a.u., respectively, whereas the corresponding magnitudes in DMIn/MMInNH_2_ and DMInNH_2_/MMIn(NH_2_)_2_ are 0.427 a.u. and 0.447 a.u. This aligns with the weaker In-N bond strength probed in TMIn:NH_3_, DMIn:NH_3_, and MMIn:NH_3_ and the stronger bond lengths in DMInNH_2_ and MMIn(NH_2_)_2_.

**Figure 4:**
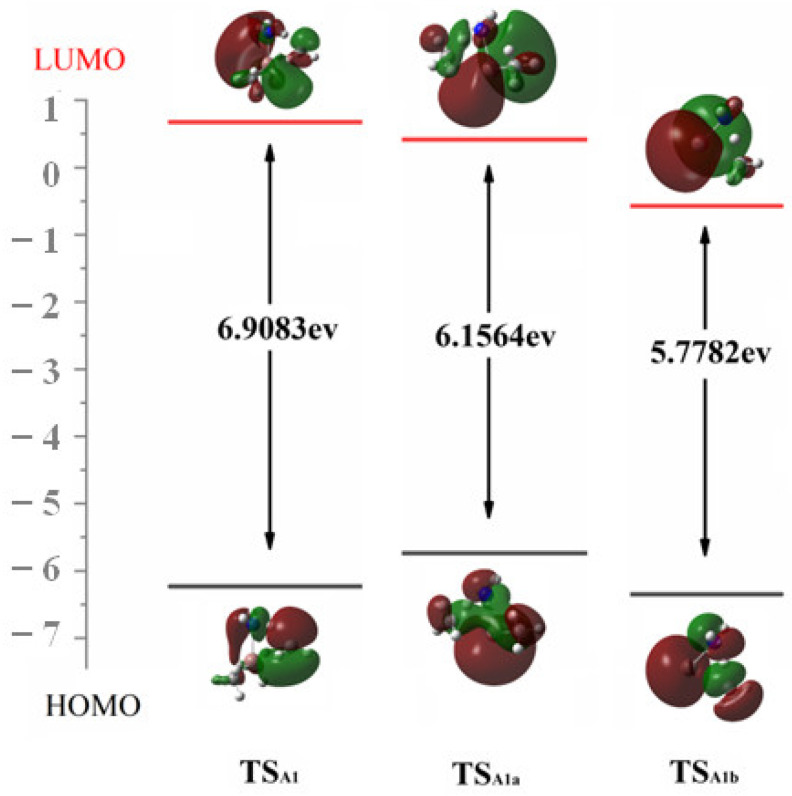
The HOMO and LUMO and the associated *E*_gap_ of TS in reactions A1, A1a and A1b.

At the M06-2X/6-311G(d,p) level of theory, the HOMO–LUMO gaps for TS_A1_, TS_A1a_ and TS_A1b_ are 6.91 eV, 6.16 eV and 5.78 eV, respectively. Although conventional DFT functions are relatively inaccurate in predicting HOMO–LUMO gaps [22], we are only concerned with the HOMO–LUMO values of different TSs obtained at the same theoretical level. This monotonic decreasing trend lines up with the *E*_a_ of the reactions. Additionally, the HOMO and LUMO of these three TSs provide valuable insights for the simultaneous NH bond cleavage. As depicted in Figure 4, during the CH bond formation in reaction A1, the HOMO suggests that the active electrons are localized between N and H, as well as the C and H. This allows them to diffuse to the *p*-like orbitals on leaving CH_3_ and facilitate CH_4_ formation. Meanwhile, the unpaired single electron in LUMO back shifts to the electron-deficient In center and hence stabilize the In-N bond.

Previous work indicates that upon being injected into the reaction chamber, XMIn (X = M, D, T) undergoes pyrolysis under high-temperature excitation and with stepwise elimination of CH_3_. These reactions are denoted as the pyrolysis path and illustrated in Table 1 (P4–P4b) [18]. Although no TS was reported in III-nitride film growth among these reactions [18,23], in this study, the PES between the predicted reactants and products was once again investigated through constrained geometry optimizations. To probe the energy change, the distance between the In and the C in TMIn was constrained at 3.75 Å, while the rest of the proposed structure was allowed to be optimized. Similar to the scans introduced above, settings were established and repeated with a 0.2 Å decrement in the step size to gradually decrease the In-C distance from 3.55 Å to 2.15 Å to mimic the pyrolysis process. As illustrated in Figure 2, no TS could be identified along the reaction coordinates. Utilizing the same method, comparable findings were obtained for pyrolysis processes P4a and P4b.

**Figure 5:**
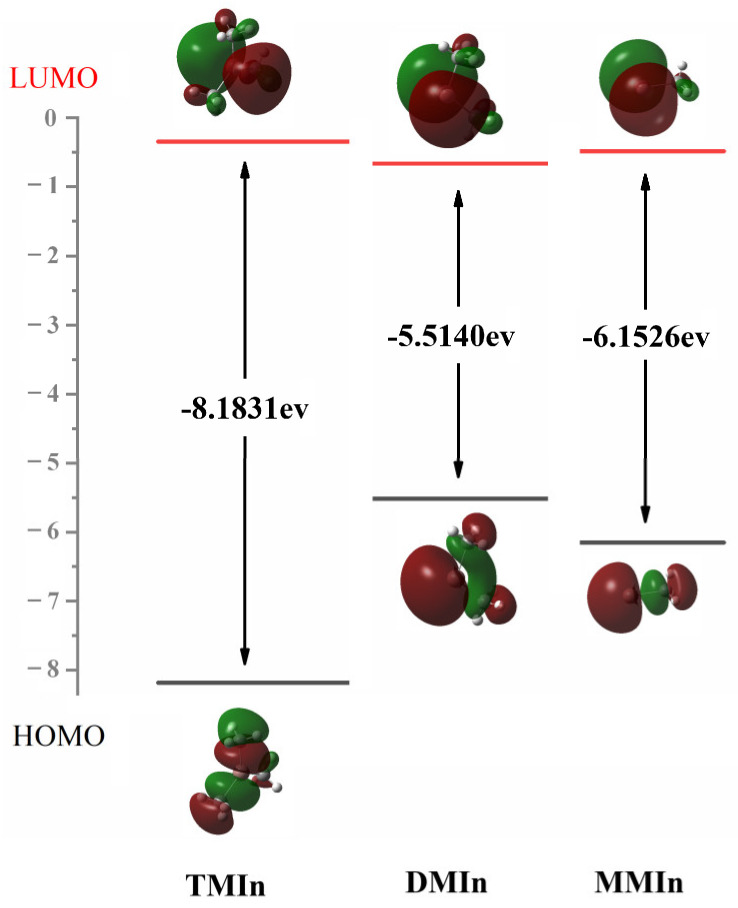
The HOMO and LUMO and the *E*_gap_ (in eV) of TMIn, DMIn and MMIn.

Also playing a critical role in the determination of the precursors for the subsequent surface reactions, XMIn pyrolysis serves as a significant alternative to the adduct route. As Section 2.1 concluded that the *BDE* trend contradicts the In-C bond length results, further NBO analysis (Table S5) was conducted on the associated three molecules to study the unique bonding in XMIn. The results indicated that the In atom of TMIn is bonded singly to three C atoms of CH_3_, resulting in a singlet ground state of TMIn. Similarly, the In atom in MMIn is bonded singly to a methyl group, so it also remains as a singlet with two electrons paired up. In contrast, in DMIn, the In atom only has one electron remaining after forming two In-C bonds with two methyl groups. This allows a DMIn doublet state. This pairing of spins leads to the significantly increased stability of singlet MMIn compared to DMIn. The FMOs for these three molecules were computed to further study the reactivity of the target molecules, and the HOMO–LUMO gaps are depicted in Figure 5. The largest HOMO–LUMO gap is observed in TMIn, followed by MMIn, while DMIn exhibits the smallest gap. Consequently, the HOMO–LUMO gap is positively correlated with the corresponding pyrolysis reaction difficulty.

#### 2.2.2. The H Radical-Involved Path

The synthesis of group III-nitride thin films via MOVPE is often complicated by the abundant radicals in the environment due to the multiple side reactions that they can initiate. Among these radicals, the H radicals can be derived from two major paths: either the homolytic H_2_ cleavage at high temperature, or the reaction between H_2_ and CH_3_ radicals which are released by the group-III precursors [19]. So, how exactly do H radicals boost XMIn pyrolysis? To address this question, all associated pathways predicted herein were thoroughly investigated. Since the high reactivity of the H radical is due to its unpaired electron, when the H radical moves close to XMIn, the electrons originally distribute between In and C will transfer to the H radical, resulting in a decrease in the strength of the In-C bond, which makes XMIn easier to pyrolyze.

The dimensionless free energy barriers reported in previous studies indicates that the ΔG*/*R*T for the pyrolysis reactions with H radicals is 39.09, 29.23 and 22.75 for TMIn, MMIn and DMIn, respectively [20]. Because the consistent trend of T > M > D (referring to the methyl groups in each compound) was also observed in reactions P4–P4b, additional investigations were conducted to confirm the role of the unpaired electron and how it can affect the reactivity of XMIn in each reaction. As illustrated in Figure S1, the H radicals are pulled close to the In atom of DMIn due to the unpaired lone electron in both. However, for TMIn and MMIn, the interaction between the H radicals and the In atoms is blocked by the methyl groups. The Mayer bond order results also show that the bond order between the H radical and the In atom of DMIn is 0.625, and 0 for both TMIn and MMIn. Based on the above analysis, more electrons shifted between DMIn and the H radical, which led to DMIn being more reactive with the H radical than TMIn and MMIn. Moreover, because the steric hindrance at the In center in TMIn is significantly larger than MMIn, the pyrolysis reaction with H radicals is more likely to occur on MMIn than on TMIn.

The occupancy of the bonds in these three TSs also provides invaluable evidence to explain why three pyrolysis reactions demand different activation energies. As listed in Table S6, the occupancy between the C atom of leaving CH_3_ in DMIn and the H radical is notably close to 2. As a significant charge transfer would occur, the electrons are shifted away from In-C when the H radical attacks the methyl group. In contrast, the corresponding occupancy between the C atom of the leaving CH_3_ in MMIn and TMIn are 0.957 and 0.910, respectively. This suggests that DMIn exhibits a greater tendency to undergo pyrolysis than TMIn and MMIn.

In addition to participating in the pyrolysis reaction of XMIn, the H radical can react with the amides DMInNH_2_ (R7) and MMInNH_2_ (R8). Rather than combining with the amino group to produce NH_3_, the H radical attacks CH_3_ and forms CH_4_ when reacting with DMInNH_2_, which may be caused by the weaker In-C bond compared to the In-N bond. Computations indicate that the In-N bond is stronger than In-C in DMInNH_2_ as the former has a *BDE* of 310.24 kJ/mol while the latter has a magnitude of 381.61. Additionally, the Mayer bond order analysis completed by Multiwfn [21] indicates that In-C has a bond order of 0.84, an In-N has a bond order of 0.91. Meanwhile, the In-C bond length (2.14 Å) is longer compared to the In-N bond length (1.98 Å). In this case, the speculated statement can be verified with a comprehensive analysis from various aspects.

Previous investigations indicate that the H radical is expected to react with the amide MMInNH_2_ through reaction R8, resulting in the formation of the intermediate MMInHNH_2_, followed by an elimination reaction and release of CH_4_ (R9) [20]. However, the lower electronic energy reported in Section 2.1 suggests that MMIn:NH_3_ could be formed prior to MMInHNH_2_. As illustrated in Figure S2, the H radical is more likely to interact with the electron-proficient N in MMInNH_2_ (rather than the In atom), leading to the production of MMIn:NH_3_ finally. Thus, this justifies our speculation. After overcoming the energy barrier, MMIn:NH_3_ may convert to MMInHNH_2_, from the Mulliken charge distribution of MMIn:NH_3_, TS_A_ in Figure 1 and MMInHNH_2_, electron transfer mainly from CH_3_ and In to NH_3_. One part of these electrons participated in the formation of the In-N bond in MMInHNH_2_. Comparing the NBO charges of the atoms in MMIn:NH_3_ (Table S4) and MMInHNH_2_ (Table S7) confirms the absence of the In-N bond in MMIn:NH_3_ and verifies the existence of a single In-N bond in MMInHNH_2_. The other part of the electrons transferred to the H atom, which is originally derived from the H radical, leading to the cleavage of the N-H bond in MMIn:NH_3_.

**Figure 6:**
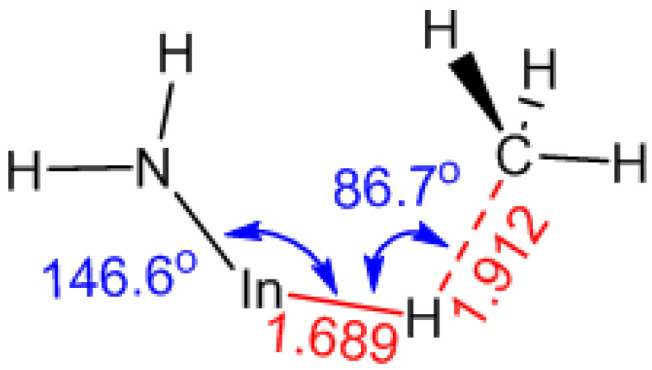
The critical bond lengths and atom distances (in Å) along with bond angles (in ^o^) in the fully optimized TS of R9.

Following the formation of MMInHNH_2_, the subsequent elimination of CH_4_ through an intramolecular reaction R9 will take place after supposing a TS as depicted in Figure 6. In this process, the In-C will be cleaved first to allow the H atom to shift to a bridging position. Then, once the high-energy complex overcomes the energy barrier of 93.62 kJ/mol, the H atom will be shifted to the methyl group, leading to the generation of CH_4_. As illustrated in Figure S3, the Mulliken charges of the In atom, the H atom from the H radical and the C atom in the reactant, MMInHNH_2_, are 0.978, −0.213, −0.839, respectively. In contrast, in the characterized TS, these values shift to 0.693, 0.060, and −0.816, respectively, suggesting that predominant charge transfer occurs from H to In.

#### 2.2.3. The H_2_ Radical-Involved Path

As outlined in Section 2.1, the interaction between the H radicals results in a considerable presence of H_2_ in the reaction environment. H_2_ is capable of reacting with XMIn (X = M, D, T) via H12–H14, thereby significantly affecting the reaction pathways. As illustrated in Figure S4, the H atom in H_2_ can be attracted by electron-proficient C in XMIn (X = M, D, T) and through the TSs depicted in Figure S5, H-H and In-C bond cleavage will facilitate CH_4_ elimination. Figure S6 compares the Mulliken charges distributed in XMIn and the associated TSs in reaction H12–H14. It is observed that when H_2_ approaches the CH_3_ group in XMIn, the charge transfer mainly occurs between the H in H_2_ during the H-H cleavage.

To elucidate the differences in *E*_a_, electron transfer was studied through Mulliken charge analysis for reactions H12–H14. Take H12 for example—excluding the leaving CH_3_ group, the total charges of the rest of the reactant and product are 0.359 a.u. and 0.219 a.u., and the sum of the charges transferred during the cleavage of In-C cleavage is 0.14 a.u. Utilizing the same method, the total charges transferred observed in H13 and H14 are 0.135 a.u. and 0.155 a.u. The trend of charge transfer follows the *E*_a_ among these three H_2_-invovled reactions and also follows the trend:H14 > H12 > H13, suggesting that the In-C bond in XMIn is more susceptible to cleavage and hence required a lower energy input to overcome the *E*_a_.

#### 2.2.4. The Amide Oligomerization Path

Nanoparticle deposition, often known as the “an unavoidable path”, presents a considerable challenge in the MOVPE growth of group III-nitrides. While numerous studies [16,18,19,23] have reported the nanoparticle compositions and elucidated their reaction thermodynamics and kinetics through both experimental and theoretical approaches, to the best of our knowledge, none have provided a comprehensive microscopic analysis of the formation and cleavage of chemical bonds in the predicted mechanisms. It is believed that nanoparticles begin to form when polymerizing the amide DMXNH_2_ (X = Al, Ga, In) [16,18,19,23]. Specifically, the amides first form cyclic dimers and trimers, which subsequently grow and increase in size via vapor-phase deposition. The reactions O10 and O11, as detailed in Table 1, correspond to the dimerization and trimerization of DMInNH_2_, respectively.

**Figure 7:**
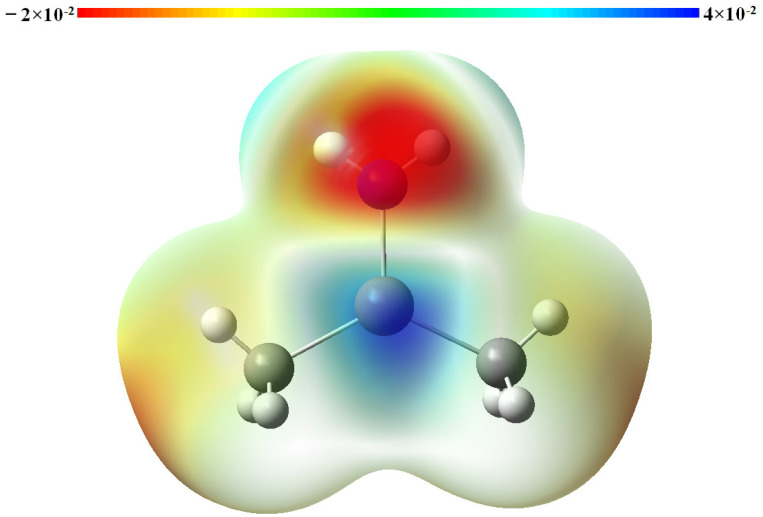
The ESP map of DMInNH_2_.

The ESP diagram of DMInNH_2_ (depicted in Figure 7) demonstrates that the electron-deficient In will interact with an electron-proficient N atom when two DMInNH_2_ molecules approach each other. This strong electrostatic interaction can initiate a nucleophilic attack: the N atom of one DMInNH_2_ molecule is prone to attacking the In atom in the neighboring DMInNH_2_ molecule, resulting in the formation of a tetragonal cyclic dimer. The Mulliken charge distribution of the monomer and the dimer (DMInNH_2_)_2_, as shown in Table 5, reveals that the In and N in DMInNH_2_ carries a charge of 1.128 a.u. and −0.913 a.u., respectively. In contrast, their Mulliken charges alter to 1.224 a.u. and −1.010 a.u., respectively, after the formation of (DMInNH_2_)_2_. Conversely, the Mulliken charge of C in DMInNH_2_ and (DMInNH_2_)_2_ remained nearly unchanged before and after the dimerization. Thus, during this process, electron transfer mainly occurs between an In atom in one DMInNH_2_ monomer and an N atom in another neighboring monomer. As demonstrated in Table 5, the charge distribution of the atoms in (DMInNH_2_)_2_ closely resembles that of the corresponding atoms in (DMInNH_2_)_3_. Thus, when DMInNH_2_ reacts with (DMInNH_2_)_2_ to generate (DMInNH_2_)_3_, the electrons mainly transfer from the In atom to the N atom intramolecularly in DMInNH_2_. The detailed NBO analyses on DMInNH_2_, (DMInNH_2_)_2_ and (DMInNH_2_)_3_ are listed in Table S8.

## 3. Computation and Methodology

All proposed structures in this study were fully characterized utilizing the density function theory (DFT) method [24] via the Gaussian 09 program package [25]. All stationary points reported herein were fully optimized using M06-2X functions [26] in conjunction with Pople’s triple-ζ split-valence basis set with polarization functions [27]. Additionally, to better describe the In atoms involved in our predicted reaction pathway, the LAN2DZ [28] basis set was applied with the effect core potential (ECP) [29] to replace the inner 46 electrons. This level of theory will be denoted as M06-2X/6-311G(d,p) and is applied for the majority of the QM computations discussed herein unless otherwise specified. The vibrational frequencies of each stationary point were computed at the same level of theory to verify its nature as either a minimum (min, n_i_ = 0) or a saddle point (TS, n_i_ = 1) on the Born–Oppenheimer potential energy surface (PES). Additionally, the TSs were verified by intrinsic reaction coordinate (IRC) [30] scans to confirm that they connect two local minima along the vibrational mode that corresponds to the only imaginary frequency.

To better calibrate the DFT baseline, we selected the most representative reaction (A1, the addition between TMIn and NH_3_ to generate the TMIn:NH_3_ adduct) among all the tested chemical processes and conducted additional quantum mechanical computations. Utilizing different methods, the reaction enthalpy change (∆*H*, in kJ/mol) was evaluated and compared with the value we reported in this paper (at the M06-2X/6-311G(d,p) level of theory). The additional computations include using ab initio methods such as the second-order Møller–Plesset perturbation theory (MP2) [31], and coupled-cluster methods where the cluster operator contains all single and double substitutions (CCSD) [32,33,34,35] along with our selected Pople’s basis set. To examine the role of dispersion functions in the selected DFT function (M06-2X), we also calculated the reaction enthalpy using the M06 function [25]. Furthermore, the ∆*H* of A1 was also determined using the popular Becke 3-parameter Lee–Yang–Parr hybrid (B3LYP) [36] function. These additional computational results are reported in Table S9. Our results indicated that without the additional correlation correction, the ∆*H* of reaction A1 is 3.98 kJ/mol higher than the value we reported in Table 1, presenting a 4.59% deviation. Compared with the popular B3LYP function, which has a large ∆*H* of −71.19 kJ/mol (with 17.78% deviation), the differences between the results obtained using the M06 and M06-2X results are less prominent. In contrast, the smallest deviation is observed when comparing the results obtained using the ab initio MP2 methods and our selected M06-2X function with a Dev_∆H_ of 2.95 kJ/mol (with 3.41% deviation). This suggests that M06-2X has the potential to provide quick yet reliable predictions when utilized with Pople’s split-valence basis set. Interestingly, larger deviations were observed when the CCSD method was applied.

For selected compounds, the crucial bond strength was determined in two different methods. The *BDE* of each stationary point anchored on the PES is determined by subtracting the electronic energy of the characterized stationary point from the sum of the two fully relaxed radical fragments (a∙ and b∙, respectively) following Equation (2).

*BDE* = *EE*_a•_ + *EE*_b•_ − *EE*_ab_ (2)

In contrast, the *E*_int_ between two molecular fragments in this project was determined based on the fully characterized stationary point. For example, for a Lewis acid and base adduct *AB*, the *E*_int_ is determined following Equation (3), where ${EE}_{A}^{SP}$ and ${EE}_{B}^{SP}$ are the single-point electronic energies of the Lewis acid and base (denoted as *A* and *B*, respectively), according to the optimized electronic structure *AB*.

(3) $$E_{\mathrm{int}}={EE}_{A}^{SP}+{EE}_{B}^{SP}-{EE}_{AB}^{\left(SP\right)}$$

## 4. Conclusions

In this study, all gas-phase reactions involved in the InN MOVPE growth proposed in previous work were rigorously revisited utilizing the DFT method at the M06-2X/6-311G(d,p) level of theory. With a comprehensive analysis of thermodynamics, ESP, NBO and Mulliken charge distribution, reaction mechanisms of pivotal gas reaction paths were conducted to elucidate the reaction mechanisms of key gas reaction pathways from a microscopic viewpoint. Based on our theoretical findings, the following conclusions can be drawn:

1. Negligible differences were observed when comparing the electronic structures optimized using double-ζ and triple-ζ split-valence basis set, the bond lengths differ by less than 0.5% and the bond angles were within a 0.85% deviation. Similarly, the same trend was also observed when analyzing the reaction energetics, as the changes in enthalpies and activation energies were within 1.2 kJ/mol and 1.3 kJ/mol, respectively. Thus, it is reasonable to believe that when applied with the M06-2X function, a double-ζ basis set can provide reliable predictions and thereby present a viable option to achieve an optimized balance between computation cost and accuracy.
2. Although it is believed that adduct formation and the XMIn pyrolysis reactions are one-step reactions that do not involve any TSs, there has been no computation verification of this assertion to the best of our knowledge. In this study, rigorous constrained geometry optimizations were utilized to scan the PES connecting the reactants and products in adduct formation (A1–A1b) and XMIn pyrolysis (P4–P4b), confirming that there are indeed no TSs for these reactions.
3. The NBO analyses verified the coordinate nature of In-N bond in TMIn:NH_3_. In contrast, computational results indicate that no bond interaction was observed between the N and the In in DMIn:NH_3_ and MMIn:NH_3_. Through a comprehensive analysis based on *E*_int_, *BDE*, Mayer bond orders and Mulliken charge transfer, the bond strength follows the trend: DMIn:NH_2_ > MMIn(NH_2_)_2_ > TMIn:NH_3_ > DMIn:NH_3_ > MMIn:NH_3_. Additionally, the Mayer bond orders of the In-N bond in TMIn:NH_3_, DMIn:NH_3_, MMIn:NH_3_, DMInNH_2_, and MMIn(NH_2_)_2_ are 0.205, 0.198, 0.142, 0.91, and 0.85, respectively, suggesting that the In–N interaction in DMInNH_2_ and MMIn(NH_2_)_2_ is close to a single bond.
4. The NBO analysis of XMIn (X = M, D, T) revealed that the valence electrons of the In atom in both TMIn and MMIn are bonded with methyl groups or have paired-up electrons, while the In in DMIn retains one unpaired electron. Thus, the complexity of XMIn pyrolysis could be attributed to electron multiplicity. During the pyrolysis of XMIn, the involvement of the highly reactive H radical facilitates electron transfer. Migration of the electrons that originally distribute between In-C bonds in XMIn cause bond cleavage and thus boost the XMIn pyrolysis.
5. In contrast to the results reported in previous work, the H radical is attracted to the electron-proficient N in MMInNH_2_, resulting in the formation of MMIn:NH_3_ rather than MMInHNH_2_. Subsequently, MMIn:NH_3_ can undergo elimination reactions and release CH_4_ through two parallel paths: one being the irreversible decomposition of A2b, and the other referred to as the “H-shift” path. In the latter, MMIn:NH_3_ can transform into MMInHNH_2_ through H-shift, followed by CH_4_ elimination via reaction R9. A comparison of the *E*_a_ for these two competing pathways indicates that the former path is the dominant one.
6. Given the substantial presence in the gas phase, H_2_ may react with the electron-proficient C in CH_3_ in XMIn (X = M, D, T) through reactions H12–H14. When undergoing these reactions, the electrons mainly transfer from one H atom to another H atom intramolecularly, leading to the cleavage of the H-H bond in H_2_. To shed light on the difference in *E*_a_ in H12–H14, the total number of electron transfers for In-C bond cleavage is calculated and the number follows the trend:H14 > H12 > H13, which aligns with the trend of *E*_a_ in H12–H14.
7. Mulliken charge distribution analysis indicated that while forming the tetragonal cyclic dimer (DMInNH_2_)_2_, intermolecular electron transfer between two DMInNH_2_ molecules predominately takes place between an In atom in the firstDMInNH_2_ and an N atom in the other. In contrast, during trimerization reaction O11, electron transfer primarily occurs intramolecularly from the In atom to the N atom within the same DMInNH_2_.

## Figures and Tables

**Table 1:** The activation energy (*E*_a_, in kJ/mol) and enthalpy change (∆*H*, in kJ/mol) of all proposed reactions.

Reaction Path	Rxn Notation	Reaction	*E*_a_ ^[a]^		∆*H* ^[a]^	
			Lit ^[b]^	6-311G(d,p)	Lit ^[b]^	6-311G(d,p)
Addition	(A1)	TMIn + NH_3_↔TMIn:NH_3_	-	-	−86.28	−86.58
	(A1a)	DMIn + NH_3_↔DMIn:NH_3_	-	-	−72.52	−73.10
	(A1b)	MMIn + NH_3_↔MMIn:NH_3_	-	-	−47.72	−47.84
	(A2)	TMIn:NH_3_→DMInNH_2_ + CH_4_	204.07	202.74	17.51	17.56
	(A2a)	DMIn:NH_3_→MMInNH_2_ + CH_4_	119.61	119.58	9.05	9.43
	(A2b)	MMIn:NH_3_→InNH_2_ + CH_4_	86.94	86.25	−36.50	−36.39
	(A3)	TMIn + NH_3_→DMInNH_2_ + CH_4_	117.78	116.16	−68.77	−69.02
	(A3a)	DMIn + NH_3_→MMInNH_2_ + CH_4_	47.09	46.48	−63.47	−63.67
	(A3b)	MMIn + NH_3_→InNH_2_ + CH_4_	39.22	38.41	−84.22	−84.23
Pyrolysis	(P4)	TMIn→DMIn + CH_3_	-	-	290.92	291.46
	(P4a)	DMIn→MMIn + CH_3_	-	-	115.92	115.68
	(P4b)	MMIn→In + CH_3_	-	-	231.81	233.09
H radical-involved	(R5)	TMIn + H→DMIn + CH_4_	68.28	67.72	−145.32	−145.63
	(R5a)	DMIn + H→MMIn + CH_4_	20.68	20.07	−320.32	−321.42
	(R5b)	MMIn + H→In + CH_4_	51.43	50.85	−204.43	−204.01
	(R6)	TMIn + H→DMInH + CH_3_	-	-	−17.60	−17.03
	(R6a)	DMIn + H→MMInH + CH_3_	-	-	−17.28	−16.72
	(R6b)	MMIn + H→InH + CH_3_	-	-	−11.75	−10.91
	(R7)	DMInNH_2_ + H→MMInNH_2_ + CH_4_	69.38	68.84	−140.02	−140.29
	(R8)	MMInNH_2_ + H→MMInHNH_2_	-	-	−313.02	−312.97
	(R9)	MMInHNH_2_→InNH_2_ + CH_4_	95.38	93.62	−28.05	−29.01
Oligomeri-zation	(O10)	2DMInNH_2_→(DMInNH_2_)_2_	-	-	−241.79	−241.90
	(O11)	(DMInNH_2_)_2_ + DMInNH_2_→(DMInNH_2_)_3_	-	-	−158.60	−158.19

^[a]^ Determined at 298.15 K and 1 atm. All basis sets were applied along with the M06-2X function. ^[b]^ Reference [20].

**Table 2:** The activation energy (*E*_a_, in kJ/mol) and enthalpy change (∆*H*, in kJ/mol) of the H_2_-involved reactions.

Reaction Path	Rxn Notation	Reaction	*E*_a_ ^[a]^		∆*H* ^[a]^	
			Lit ^[b]^	6-311G(d,p)	Lit ^[b]^	6-311G(d,p)
H_2_-involved	(H12)	TMIn + H_2_→DMInH + CH_4_	148.60	148.51	−24.66	−24.60
	(H13)	DMIn + H_2_→MMInH + CH_4_	150.17	150.04	−24.34	−24.29
	(H14)	MMIn + H_2_→InH + CH_4_	120.23	120.23	−18.81	−18.48

^[a]^ Determined at 298.15 K and 1 atm. All basis sets were applied along with the M06-2X function. ^[b]^ Reference [20].

**Table 3:** NBO charges on TMIn:NH_3_ at M06-2X/6-311G(d,p) level of DFT theory.

Occupancy	Bond Orbital		Hybdrids			
1.99	C-H	60.15%	C	*s* (24.73%)	*p* (75.21%)	*d* (0.06%)
		39.85%	H	*s* (99.95%)	*p* (0.05%)	-
1.99	C-In	81.56%	C	*s* (25.84%)	*p* (75.16%)	*d* (0.00%)
		18.44%	In	*s* (29.63%)	*p* (70.37%)	-
1.98	In-N	5.00%	In	*s* (11.10%)	*p* (88.90%)	-
		95.00%	N	*s* (31.82%)	*p* (68.16%)	*d* (0.02%)
1.99	N-H	69.53%	N	*s* (22.69%)	*p* (77.23%)	*d* (0.08%)
		30.47%	H	*s* (99.94%)	*p* (0.06%)	-

**Table 4:** The *BDE* values of the In-N bond (in kJ/mol), Mayer bond order and the Mulliken charge transfer (*N*_B→A_, in Atomic Units) between In and N in XMIn:NH_3_ and XMIn(NH_2_)_Y_ where X = M, D, T and Y = 1, 2.

Molecule	*BDE*	Mayer Bond Order	*N*_B→A_
TMIn:NH_3_	92.76	0.205	0.126
DMIn:NH_3_	80.03	0.198	0.122
MMIn:NH_3_	54.05	0.142	0.082
DMInNH_2_	381.61	0.91	−0.427
MMIn(NH_2_)_2_	370.57	0.85	−0.447

Annotation: The positive charge transfer means from the N-involved segment to the In-involved segment and vice versa.

**Table 5:** Mulliken charge distribution of DMInNH_2_, (DMInNH_2_)_2_ and (DMInNH_2_)_3_.

Molecule	In	N	C
DMInNH_2_	1.128	−0.913	−0.831
(DMInNH_2_)_2_	1.224	−1.010	−0.834
(DMInNH_2_)_3_	1.258	−1.015	−0.800

## Data Availability

Data are contained within the article and Supplementary Materials.

## Supplementary Materials

The following supporting information can be downloaded at: https://www.mdpi.com/article/10.3390/molecules30040971/s1. Table S1. A comparison of selected bond lengths (in Å) of pivotal molecules analyzed in this study; Table S2. Comparison of selected bond angles (in °) of pivotal molecules computed in this study; Table S3. Nature bond orbitals of DMIn:NH_3_; Table S4. Nature bond orbitals of MMIn:NH_3_; Table S5. Nature bond orbitals of In-C bond of XMIn (X = M, D, T); Table S6. The occupancy of the bonds in characterized TSs in H radical-involved pyrolysis reaction path; Table S7. Nature bond orbitals of MMInHNH_2_; Table S8. Nature bond orbitals of DMInNH_2_, (DMInNH_2_)_2_ and (DMInNH_2_)_3_; Table S9. The enthalpy change (∆*H*, in kJ/mol) of reaction A1 (TMIn+NH_3_↔TMIn:NH_3_) and the enthalpy change deviation (Dev_∆H_, in kJ/mol) with its percent deviation results obtained at different ab initio and DFT levels of theory; Figure S1. The optimized structure of TSs in H-involved XMIn (X = M, D, T) pyrolysis reaction; Figure S2. The ESP map of MMInNH_2_; Figure S3. The Mulliken charge distribution of MMInHNH_2_ and corresponding TS in reaction R9; Figure S4. The ESP map of H_2_ and XMIn (X = M, D, T); Figure S5. Structure parameters in XMIn (X = M, D, T) and corresponding XMIn + H_2_ TS; Figure S6. Mulliken charge on the key atoms in XMIn (X = M, D, T) and corresponding XMIn + H_2_ TS.
