# Supplementary material for: Revisiting the Mechanistic Pathway of Gas-Phase Reactions in InN MOVPE Through DFT Calculations
Source: Molecules. 2025 Feb 19;30(4):971. doi: 10.3390/molecules30040971 (PMC11857898; doi:10.3390/molecules30040971)
Supplement: Supplementary file 1 [file molecules-30-00971-s001.zip › molecules-3469977-supplementary.pdf]

Revisiting the Mechanistic Pathway of Gas-Phase Reactions in InN MOVPE Through DFT Calculations

Supplementary Information

Xiaokun He [1,2], Nan Xu [3], Yuan Xue [4,5,*], Hong Zhang [6], Ran Zuo [6] and Qian Xu [2,*]

([1] Suzhou Institute of Technology, Jiangsu University of Science and Technology, Zhangjiagang 215600, China; kenhe25@163.com

[2] Institute for Energy Research, Jiangsu University, Zhenjiang 212013, China

[3] State Key Laboratory of High-Efficiency Utilization of Coal and Green Chemical Engineering, Faculty of Advanced Science and Technology, Ningxia University, Yinchuan 750021, China; nan.xu@nxu.edu.cn

[4] Department of Chemistry and Biochemistry, The University of Mississippi, Oxford, MS 38677, USA

[5] Department of Chemistry, The University of Akron, Akron, OH 44325, USA

[6] School of Energy and Power Engineering, Jiangsu University, Zhenjiang 212013, China;

thornbird05@126.com (H.Z.); rzocean111@163.com (R.Z.)

***Corresponding authors**

(E-mail addresses: yxue@olemiss.edu (Y.X.); xuqian@ujs.edu.cn (Q.X.))

Table of Contents

1. Table S1……………………………………………………………...P.1

2. Table S2……………………………………………………………...P.1

3. Table S3……………………………………………………………...P.1

4. Table S4…………………………………………………………...…P.2

5. Table S5……………………………………………………....….…..P.2

6. Table S6……………………………………………………....….…..P.2

7. Table S7……………………………………………………....….…..P.2

8. Table S8……………………………………………………....….…..P.3

9. Table S9……………………………………………………....….…..P.3

10. Figure S1…………………………………………….…..... …....P.4

11. Figure S2……………………………………………………..…....P.4

12. Figure S3……………………………………………………....…..P.4

13. Figure S4……………………………………………………....…..P.5

14. Figure S5……………………………………………………....…..P.6

15. Figure S6……………………………………………………....…..P.7

Table S1. A comparison of selected bond lengths(in Å) of pivotal molecules analyzed in this study

Molecule	Bond Type	Bond length	
Pyrolysis path related			
**Molecule**	**Bond Type**	**M06-2X/6-31G(d,p)**	**M06-2X/6-311G(d,p)**
TMIn	In-C	2.15	2.15
	C-H	1.09	1.09
DMIn	In-C	2.19	2.19
	C-H	1.09	1.09
Adduct path related			
**Molecule**	**Bond Type**	**Bond length**	
		**M06-2X/6-31G(d,p)**	**M06-2X/6-311G(d,p)**
TMIn:NH_3	In-C	2.17	2.17
	C-H	1.10	1.09
	In-N	2.39	2.40
DMInNH_2	In-C	2.14	2.14
	C-H	1.09	1.09
	In-N	1.98	1.98
Oligomerization path related			
**Molecule**	**Bond Type**	**Bond length**	
		**M06-2X/6-31G(d,p)**	**M06-2X/6-311G(d,p)**
$(DMInNH_2)_2$	In-N	2.21	2.21
	C-In	2.15	2.15
$(DMInNH_2)_3$	In-N	2.20	2.20
	C-In	2.15	2.16

Table S2. Comparison of selected bond angles (in °) of pivotal molecules computed in this study

Bond Angle	Molecule	Bond angles	
		**M06-2X/6-31G(d,p)**	**M06-2X/6-311G(d,p)**
$\angle_{C\text{-}In\text{-}C}$	TMIn	120.00	120.00
	DMIn	117.47	116.41
	TMIn:NH_3	118.81	118.88
	DMInNH_2	126.46	125.99
$\angle_{C\text{-}In\text{-}N}$	TMIn:NH_3	96.27	96.12
	DMInNH_2	116.77	117.00
$\angle_{In\text{-}N\text{-}In}$	$(DMInNH_2)_2$	96.20	96.31
	$(DMInNH_2)_3$	114.68	114.63
$\angle_{N\text{-}In\text{-}N}$	$(DMInNH_2)_2$	83.80	83.80
	$(DMInNH_2)_3$	96.90	96.68

Table S3. Nature bond orbitals of DMIn:NH_3

Spin	Occupancy	Bond orbital		Hybdrids			
α	0.99	C-H	59.77%	C	*s* (23.47%)	*p* (76.46%)	*d* (0.07%)
			40.23%	H	*s* (99.95%)	*p* (0.05%)	
β	0.99	C-H	60.19%	C	*s* (27.33%)	*p* (72.61%)	*d* (0.06%)
			39.81%	H	*s* (99.95%)	*p* (0.05%)	
α	0.99	C-In	85.23%	C	*s* (25.97%)	*p* (74.03%)	*d* (0.00%)
			14.77%	In	*s* (17.39%)	*p* (82.61%)	
β	0.98	C-In	77.04%	C	*s* (20.97%)	*p* (79.03%)	*d* (0.01%)
			22.96%	In	*s* 48.67%)	*p* (51.33%)	
α	0.99	N-H	69.30%	N	*s* (26.20%)	*p* (73.71%)	*d* (0.08%)
			30.70%	H	*s* (99.94%)	*p* (0.06%)	
β	0.99	N-H	69.20%	N	*s* (26.20%)	*p* (73.71%)	*d* (0.08%)
			30.80%	H	*s* (99.94%)	*p* (0.06%)	

Table S4. Nature bond orbitals of MMIn:NH$_3$

Occupancy	Bond orbital		Hybdrids			
1.99	C-H	59.63%	C	*s* (25.08%)	*p* (74.85%)	*d* (0.07%)
		40.37%	H	*s* (99.95%)	*p* (0.05%)	
1.99	C-In	88.39%	C	*s* (23.59%)	*p* (76.41%)	*d* (0.00%)
		11.61%	In	*s* (8.74%)	*p* (91.26%)	
1.99	N-H	69.53%	N	*s* (25.76%)	*p* (74.16%)	*d* (0.09%)
		30.47%	H	*s* (99.93%)	*p* (0.07%)	

Table S5. Nature bond orbitals of In-C bond of XMIn (X=M, D, T)

Molecule	Spin	Occupancy	Bond orbital		Hybdrids			
TMIn	-	1.96	C-In	80.70%	C	*s* (23.52%)	*p* (76.47%)	*d* (0.01%)
				19.30%	In	*s* (33.33%)	*p* (66.67%)	
DMIn	α	0.99	C-In	86.24%	C	*s* (23.27%)	*p* (72.72%)	*d* (0.06%)
				13.76%	In	*s* (13.80%)	*p* (86.20%)	
	β	0.97	C-In	74.44%	C	*s* (18.21%)	*p* (81.78%)	*d* (0.00%)
				25.56%	In	*s* (49.66%)	*p* (50.34%)	
MMIn	-	1.99	C-In	88.50%	C	*s* (20.72%)	*p* (79.28%)	*d* (0.00%)
				11.50%	In	*s* (7.60%)	*p* (92.40%)	

Table S6. The occupancy of the bonds in characterized TSs in H radical-involved pyrolysis reaction path

TS	In-C	C-H radical
TMIn_H	0.942	0.910
DMIn_H	-	1.752
MMIn_H	0.992	0.957

Table S7. Nature bond orbitals of MMInHNH$_2$

Occupancy	Bond orbital		Hybdrids			
1.99	C-H	60.91%	C	*s* (25.48%)	*p* (74.46%)	*d* (0.06%)
		39.09%	H	*s* (99.95%)	*p* (0.05%)	
1.97	C-In	79.09%	C	*s* (23.05%)	*p* (76.94%)	*d* (0.01%)
		20.91%	In	*s* (36.60%)	*p* (63.40%)	
2.00	In-N	6.43%	In	*s* (0.00%)	*p* (100.00%)	
		93.57%	N	*s* (0.00%)	*p* (100.00%)	*d* (0.00%)
1.98	In-N	12.85%	C	*s* (30.11%)	*p* (69.89%)	
		87.15%	In	*s* (42.89%)	*p* (57.10%)	*d* (0.00%)
1.96	In-H	27.41%	In	*s* (33.44%)	*p* (66.56%)	
		72.59%	H	*s* (99.93%)	*p* (0.07%)	
2.00	N-H	68.55%	N	*s* (28.37%)	*p* (71.56%)	*d* (0.07%)
		31.45%	H	*s* (99.94%)	*p* (0.06%)	

Table S8. Nature bond orbitals of $DMInNH_2$, $(DMInNH_2)_2$ and $(DMInNH_2)_3$

Molecule	Occupancy	Bond orbital		Hybdrids			
$DMInNH_2$	1.99	C-H	60.82%	C	*s* (25.46%)	*p* (75.48%)	*d* (0.06%)
			39.18%	H	*s* (99.95%)	*p* (0.05%)	
	1.99	C-In	80.06%	C	*s* (23.03%)	*p* (76.96%)	*d* (0.01%)
			19.94%	In	*s* (35.39%)	*p* (64.61%)	
	1.99	In-N	6.04%	In	*s* (0.00%)	*p* (100.00%)	
			93.96%	N	*s* (0.00%)	*p* (100.00%)	*d* (0.00%)
	1.98	In-N	12.31%	In	*s* (29.25%)	*p* (70.75%)	
			87.69%	N	*s* (42.99%)	*p* (57.00%)	*d* (0.01%)
	1.99	N-H	68.50%	N	*s* (28.44%)	*p* (71.50%)	*d* (0.07%)
			31.50%	H	*s* (99.94%)	*p*(0.06%)	
$(DMInNH_2)_2$	1.99	C-H	60.66%	C	*s* (24.95%)	*p*(74.99%)	*d* (0.06%)
			39.34%	H	*s* (99.95%)	*p*(0.05%)	
	1.99	C-In	80.74%	C	*s* (24.94%)	*p* (75.05%)	*d* (0.01%)
			19.26%	In	*s* (31.37%)	*p* (68.63%)	
	1.99	In-N	8.72%	In	*s* (18.63%)	*p* (81.37%)	
			91.28%	N	*s* (27.57%)	*p* (72.43%)	*d* (0.00%)
	1.99	N-H	69.15%	N	*s* (22.38%)	*p* (77.54%)	*d* (0.08%)
			30.85%	H	*s* (99.94%)	*p* (0.06%)	
$(DMInNH_2)_3$	1.99	C-H	60.17%	C	*s* (24.18%)	*p* (75.76%)	*d* (0.06%)
			39.83%	H	*s* (99.95%)	*p* (0.05%)	
	1.99	C-In	81.72%	C	*s* (25.76%)	*p* (74.24%)	*d* (0.01%)
			18.28%	In	*s* (30.90%)	*p* (69.10%)	
	1.99	In-N	8.46%	In	*s* (18.57%)	*p* (81.43%)	
			91.54%	N	*s* (29.49%)	*p* (70.50%)	*d* (0.01%)
	1.99	N-H	69.14%	N	*s* (20.47%)	*p* (79.44%)	*d* (0.09%)
			30.86%	H	*s* (99.95%)	*p* (0.05%)	

Table S9. The enthalpy change (ΔH, in kJ/mol) of reaction A1 ($TMIn+NH_3 \leftrightarrow TMIn{:}NH_3$) and the enthalpy change deviation ($Dev_{\Delta H}$, in kJ/mol) with its percent deviation results obtained at different *ab initio* and DFT levels of theory

Level of theory	ΔH[a]	$Dev_{\Delta H}$ (%)[b]
M06-2X/6-311G(d,p)	-86.58	-
M06/6-311G(d,p)	-82.60	+3.98 (4.59 %)
B3LYP/6-311G(d,p)	-71.19	+15.39 (17.78%)
MP2/6-311G(d,p)	-83.63	+2.95 (3.41%)
CCSD/6-311G(d,p)	-77.47	+9.11 (10.52%)

[a] Determined at 298.15K and 1 atm.

[b] Relative to the results obtained at M06-2X/6-311G(d,p) level of theory.

Determined using the equation:

$$\%Dev_{\Delta H} = \frac{Dev_{\Delta H}}{\Delta H_{M06-2X\,/\,6-311G(d,p)}} \times 100\%$$

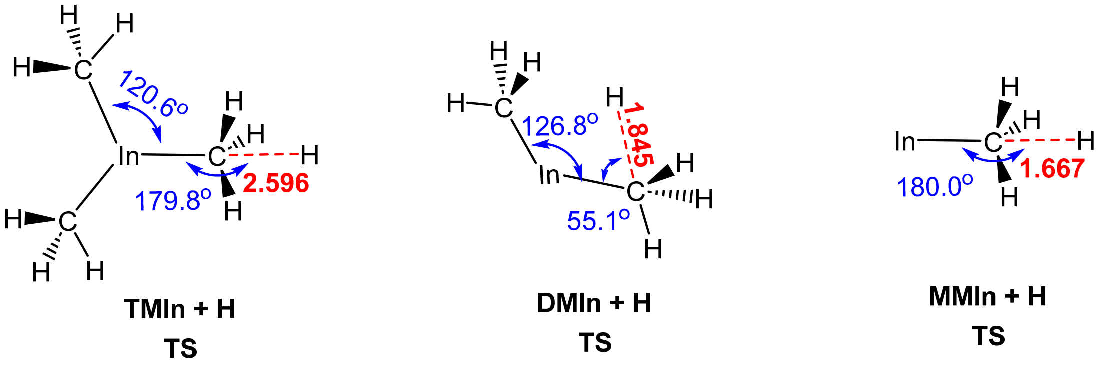

Figure S1. The optimized structure of TSs in H-involved XMIn (X=M, D, T) pyrolysis reaction

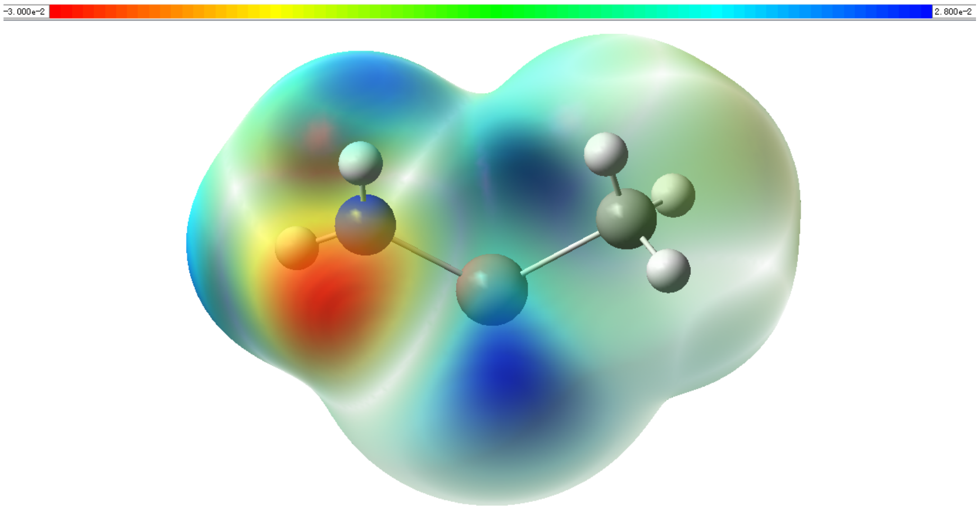

Figure S2. The ESP map of $MMInNH_2$

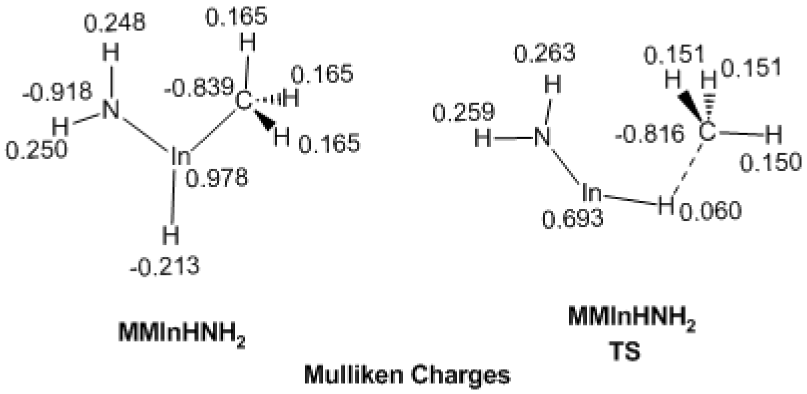

Figure S3. The Mulliken charge distribution of $MMInHNH_2$ and corresponding TS in reaction R9

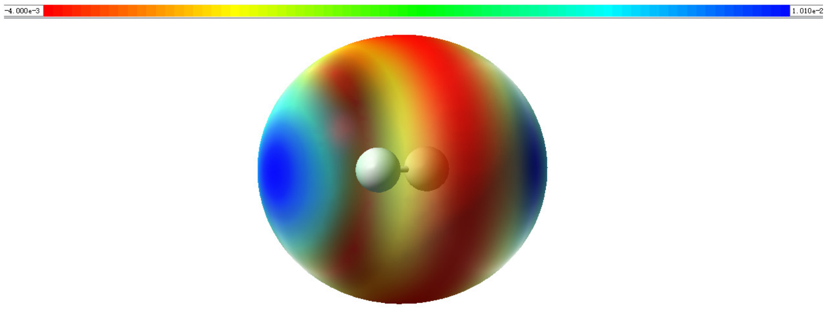

H_2

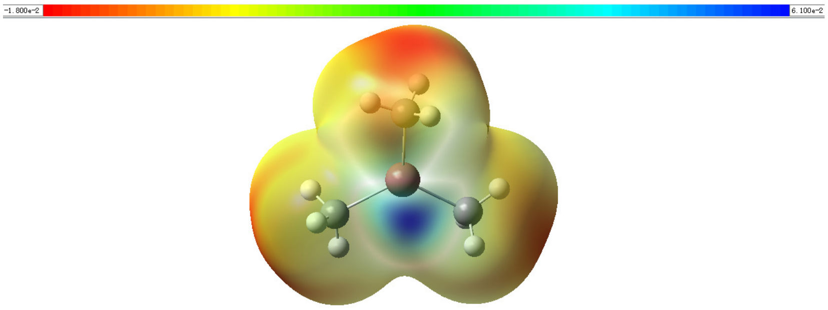

TMIn

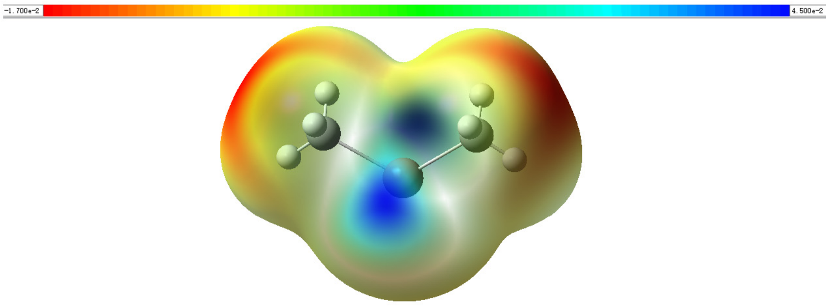

DMIn

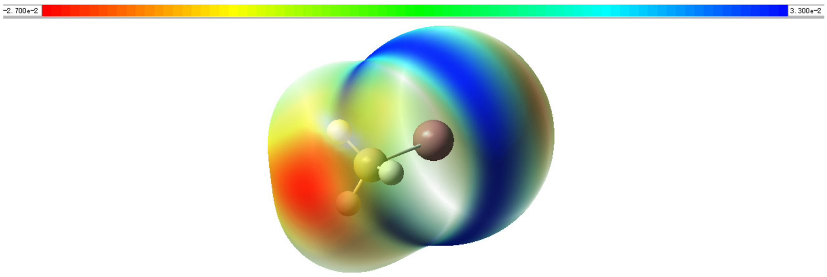

MMIn

Figure S4. The ESP map of H_2 and XMIn (X=M, D, T)

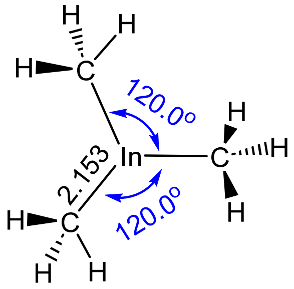

TMIn

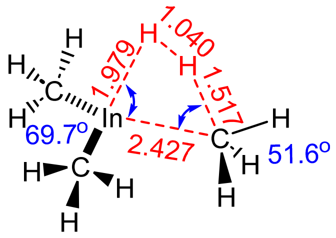

TMIn + H_2 TS

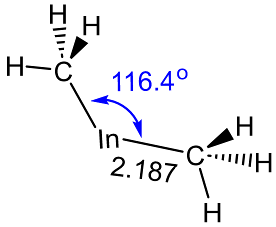

DMIn

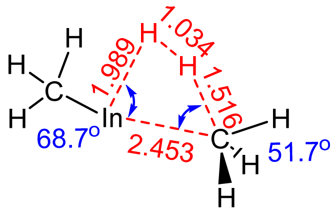

DMIn + H_2 TS

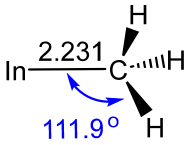

MMIn

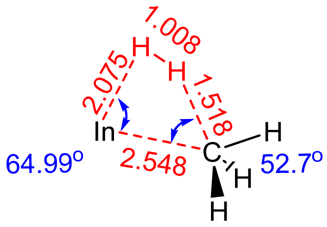

MMIn + H_2 TS

Figure S5. Structure parameters in XMIn (X=M, D, T) and corresponding XMIn+H_2 TS

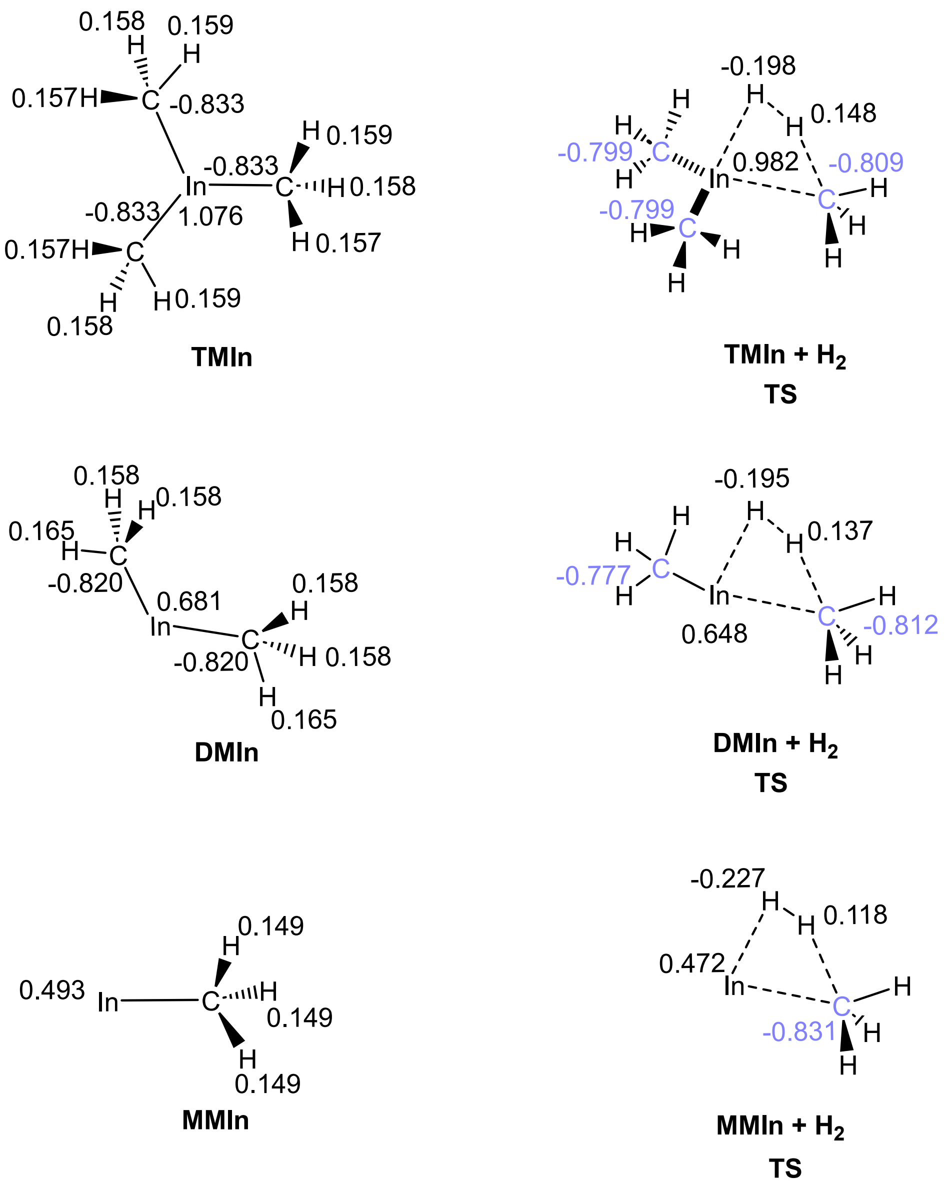

Figure S6. Mulliken charge on the key atoms in XMIn (X=M, D, T) and corresponding XMIn+H_2 TS
